# Recent advances in the understanding of tubal ectopic pregnancy

**DOI:** 10.12703/r/12-26

**Published:** 2023-11-01

**Authors:** Heather C Flanagan, W Colin Duncan, Chih-Jen Lin, Norah Spears, Andrew W Horne

**Affiliations:** 1 MRC Centre for Reproductive Health, University of Edinburgh, Edinburgh, Scotland; 2 Biomedical Sciences, University of Edinburgh, Edinburgh, Scotland

**Keywords:** Ectopic pregnancy, risk factors, clinical treatment, ultrasound, laparoscopic salpingectomy, methotrexate

## Abstract

Ectopic pregnancy (EP) is described as the implantation of an embryo outside the normal uterine cavity. It most commonly occurs in the fallopian tube, hence termed a tubal ectopic pregnancy (tEP). It is a gynaecological emergency and remains the leading cause of direct maternal mortality related to the first trimester of pregnancy worldwide. This article explores the emergence of additional risk factors for tEP, showing new evidence for identifying patient risk factors and highlighting potential areas of research. Additionally, we discuss the up-to-date patient-centred approach for the diagnosis, management and counselling of patients with tEP and ongoing clinical trials for the improvement of medical management.

## Introduction

An ectopic pregnancy (EP) is a gynaecological emergency and is described as the implantation of an embryo outside the normal uterine cavity^1^. EP occurs in 1–2% of all pregnancies and can cause significant intraperitoneal bleeding. It is the most common early pregnancy-related cause of maternal morbidity and mortality^2,3^. The most common site of extra-uterine implantation is the Fallopian tube, termed a tubal ectopic pregnancy (tEP), which accounts for 95–98% of EPs. Other sites of implantation include the ovary, cervix, abdominal cavity and caesarean section scars^2^. Very rarely, EP can occur in other areas - for example, in an underdeveloped half of the uterus called the rudimentary horn or in the myometrium (termed an intramural EP). A heterotopic pregnancy occurs when an embryo implants in the uterine cavity with simultaneous implantation of another embryo outside of the cavity^1^.

## Impacts of tubal ectopic pregnancy

### Morbidity

Short-term morbidity for tEP can be from haemorrhagic shock and anaemia or due to complications arising from clinical management of the tEP - for example, side effects of medical management^1^, venous thromboembolism or infection associated with surgical intervention. Long-term morbidity for EP includes a reduction in fertility, increased chance of tEP recurrence and an impact on mental health, with 23% of women meeting the criteria for post-traumatic stress disorder (PTSD)^4^. PTSD increases the risk of spontaneous abortion, preterm delivery and low birth weight^5,6^. Additionally, work, social interaction and the utilisation of health care can be affected by PTSD, and this ultimately negatively impacts overall health and wellbeing^1,4^.

There is an emerging association of increased risk for ovarian carcinoma with previous tEP. Novel findings of an increased risk of serous borderline ovarian tumour associated with prior tEP have been reported^7^. However, it is hypothesised that this increased risk may be due to other confounding factors, such as pelvic inflammatory disease associated with tEP^7^. Another study found that the surgical removal of a fallopian tube for ectopic pregnancy reduced the incidence of ovarian cancer; however, there was no protective effect in the first years following the removal of a fallopian tube, suggesting that there may be a lag-time for protective effect after intervention^8^. The identification of a possible interaction between ectopic pregnancy and ovarian serous borderline tumours further supports the hypothesis that ovarian cancer originates in the fallopian tube^7^. It is important to note that evidence behind this association is limited and further research is needed. 

### Mortality

In high-income developed countries, the death rate for women diagnosed with a tEP remains relatively low, with a mortality ratio of 0.4 per 100,000 live births in the UK and 0.5 per 100,000 live births in the US^9,10^. In high-income countries, there are health disparities with worse outcomes associated with some ethnic minority groups or those with low-income. The mortality rate is much higher in low-middle income developing countries; for example, in Brazil, the mortality ratio for tEP is 1.2 per 100,000 live births^11^. In resource-poor developing countries in Africa, the mortality ratio for tEP is not clearly defined but is thought to be significantly higher than in developed countries^12^. This highlights the importance of effective sexual health services and appropriate resources for early diagnosis and management, as well as standardisation of care^13^. 

An enquiry into maternal deaths in the UK in 2019 by ‘Mothers and Babies: Reducing Risk through Audits and Confidential Enquiries across the UK’ (MBRRACE-UK) showed no decrease in tEP-related maternal deaths since the 2016 report, which revealed that 4.8% of all direct maternal deaths were related to tEP. In the 2012–14 MBRRACE report, it was shown that other diagnoses were suspected in 5 out of the 9 cases and that earlier consideration of tEP could have prevented some of these deaths^14^. In addition, in the 2019 report, delays in emergency care and/or transfer to hospital were reported to have been involved with all five cases of tEP-related deaths^3^. It is important to consider that MBRRACE reports exclude some key clinical data for confidentiality reasons to allow for a more detailed assessment of clinical antecedents. Ultimately, early diagnosis and treatment for tEP are key for reducing morbidity and mortality, and those presenting with symptoms of a ruptured tEP should have timely emergency care.

## Risk factors

Although there are well-documented risk factors for ectopic pregnancy, most women have no significant risks that could predict an increased risk of tEP. However, it is important to assess risk factors since the incidence of EP is increased by several factors^2^, including tubal damage, the presence of an intrauterine device, maternal age, smoking, assisted reproductive technologies (ART), air pollution, pelvic inflammatory disease and inflammatory bowel disease (IBD)^1,15,16^. Generally, these risk factors are conditions that are associated with changes in the structure or function of the fallopian tube. An understanding of the risk factors is important as it can be used to help triage patients to facilitate rapid diagnosis.

### Tubal damage

Tubal damage is a major risk factor for tEP, accounting for up to a third of all tEP cases. It can be the result of surgery, previous infection or previous tEP^2,17^. Previous tEP represents the biggest risk factor for recurrent tEPs. A controlled follow-up study found that women whose first pregnancy was ectopic had an adjusted odds ratio (OR) of 4.7 - 10.0 for increased risk of recurrence^18^. The risk increases greatly with the number of prior tEPs up to an OR of 17.16^19^. Most of the data on risk of EP after previous tEP is based on data linkage studies and focussed on surgical management. Studies looking at the effect of methotrexate or medical management where the tube is left behind do not show any extra risk of ectopic pregnancy - if anything, the risk was lower than after salpingectomy^20,21^.

Tubal surgery, including tuboplasty, salpingostomy, re-anastomosis and adhesiolysis, can also increase the risk of developing a tEP. It has been reported that tubal surgery can result in incidences of tEP of up to 40% depending on the severity of the damage; however, one study reported that tEP risk after salpingostomy and adhesiolysis was around 7.9% and after re-anastomosis was 6.7%^22^.

### Maternal age

Maternal age has been associated with a higher risk for the occurrence of tEP; the increase in OR of ectopic pregnancy in advanced maternal age groups (≥44) has been reported to be 6.9^23,24^. The increasing risk of tEP with maternal age differs from miscarriage. The rate of miscarriage increasing with maternal age is mainly due to the increase in chromosomal abnormalities in the embryo; however, this has been ruled out as a cause of tEP^25^. While the reason for the observed increase remains unknown, it is hypothesised that age can cause impaired tubal function, including delayed transport of the embryo to the uterus^26^.

### Cigarette smoking

**Figure 1.**
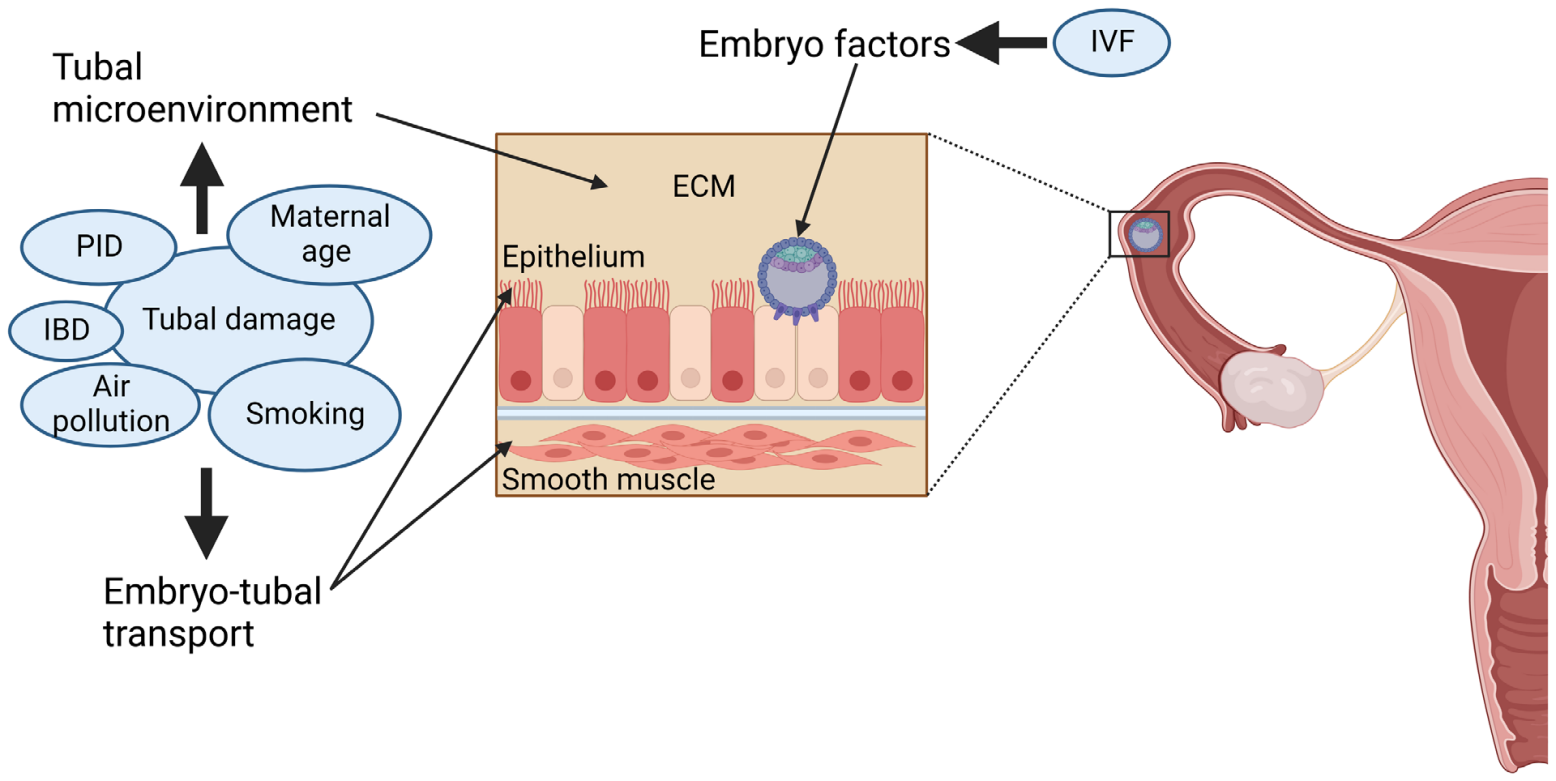
Risk factors for ectopic pregnancy. The risk associated with tubal damage, maternal age, smoking, air pollution, Pelvic inflammatory disease (PID) and inflammatory bowel disease (IBD) is due to effects on tubal microenvironment and on embryo-tubal transport: the tubal microenvironment is affected by changes to the extracellular matrix (ECM), while effects on smooth muscle and epithelial cilia function alter embryo-tubal transport. The risk associated with IVF is caused by embryo factors. The size of risk factors in the figure indicates relative contribution to increased risk.

Cigarette smoking is a major risk factor for tEP, but the cause of this increased risk remains largely unknown^27^. Cigarette smoke contains over 4000 chemicals. Cotinine, which is the active component in nicotine, has been the only cigarette smoke component studied to date in tEP research. Cotinine has been shown to cause changes to embryo transport, embryo-tubal interactions and the tubal microenvironment (Figure 1)^27,28^. This would imply that vaping may increase the risk of tEP, but to date, that has not been studied. However, a major limitation is that these studies have focussed on only one component of cigarette smoke. Studies focusing on cigarette smoke components that are known to induce cellular changes, such as Benzo(a)Pyrene, are needed to understand the full range of effect cigarette smoke may have on the fallopian tube epithelial layer. The OR for tEP in current smokers has recently been calculated to be 4.21^19^.

### Assisted reproductive technologies

Assisted reproductive technologies (ART), such as *in vitro* fertilisation (IVF), are risk factors for tEP^29^. The reported rates of tEP after IVF range from 1.6–8.9%, but it is challenging to separate IVF itself from the reasons that IVF was required. Adjusted risk ratios for EP during IVF are variable^29^. The highest risk of tEP during IVF was associated with tubal factor infertility (OR 3.9); however, a decreased risk (OR 0.6) was associated with the endometrial combined thickness (ECT) of >12mm^29^. Recently, studies have identified prognostic factors for EP following ART, including fresh embryo transfer, the use of GnRH agonists, number of embryos transferred in a cycle, transfer depth and volume of transfer fluid^2,30,31^. It has also been reported that embryo transfer during the cleavage stage has significantly higher rates of tEP compared to transfer at the blastocyst stage^29^. This suggests that IVF itself is likely to be an independent risk factor (Figure 1). However, because tEP after IVF represents a failure to implant in the endometrium, it has been hypothesised that differences in the hormonal or molecular environment of the uterus may be responsible for the increased risk of tEP associated with IVF^29^.

### Ambient air pollution

Recently, there has been growing concern about air pollution and adverse pregnancy outcomes such as spontaneous abortion, preterm birth, intrauterine growth restriction and stillbirth^32,33^. New evidence suggests a significant association between exposure to air pollution and an increased risk of tEP, suggesting that air pollution increases tEP risk in IVF patients, with an adjusted OR of 2.68^16^. The increased risk associated with air pollution further highlights that there are likely elements of cigarette smoke outside nicotine that increase the risk of tEP. Some limitations of these studies exist. For example, the lack of information on workplace addresses and socio-economic confounders were not adjusted in the analysis, suggesting that further analysis is necessary to understand the full extent of the effect of air pollution on the risk of ectopic pregnancy^16^.

### Pelvic inflammatory disease

A history of pelvic infection or pelvic inflammatory disease (PID), particularly after infection with *Chlamydia trachomatis* (CT), is a well-known contributor to tubal damage associated with an increased risk of subsequent tEP^2^. In those with previous CT, the incidence of tEP is reported to be 1.2 in 1000^34^. The risk of tEP increases with repeated infection; in ascending infections, salpingitis (inflammation of the Fallopian tube) can develop, and tubal damage occurs, leading to tubal dysfunction and aberrant embryo implantation^2^. Furthermore, CT infection and subsequent PID have been associated with an increased risk of ovarian cancer, suggesting that CT may induce epithelial-mesenchymal transition (EMT) in the fallopian tube, resulting in an altered tubal microenvironment (Figure 1)^35^. PID has been reported to increase the risk of tEP to an OR of 2.1 (Table 1)^36^.

**Table 1.** Adjusted risk ratios for EP risk factors.

*Risk factor*	Adjusted OR	Adjusted RR	Increased risk (%)
*Previous ectopic pregnancy*	17.16	12.96	1196
*Tubal surgery*	2.6 - 7.9	2.52 - 6.94	152 - 594
*Age (≥44 years)*	6.9	6.17	517
*Smoking*	4.21	3.95	295
*IVF*	0.6 - 3.9	0.6 - 3.68	40 - 268
*Ambient air pollution*	2.68	2.59	159
*Pelvic inflammatory disease*	2.1	2.05	105
*IBD*	1.1 - 1.49	1.09 - 1.47	9 - 47

Table 1 shows the adjusted odds ratios (OR) and adjusted risk ratio (RR) (converted from OR based on overall prevalence of EP) for each known risk factor in order of diminishing risk. The adjusted risk ratios were then calculated relative to ectopic pregnancy risk without any risk factors to give the percentage increased risk according to BMJ best practice toolkit. Table adapted from Farquhar, C. (2005).

### Inflammatory bowel disease

A newly emerged risk factor for tEP is inflammatory bowel disease (IBD). Until recently, there has been limited data on IBD and tEP risk due to small sample sizes^15^. A population-based cohort study found that women with Crohn’s disease were at higher relative risk of tEP than pregnancies in women without IBD, although the risk is still low (Table 1). Patients who had undergone surgery for IBD were suggested to have a higher risk for tEP. Outside surgical adhesions, the increased risk for EP observed in IBD patients could be in part due to the pathology of IBD, as patients are more likely to have perforating and fistulising pathology with non-surgical adhesions^15^. Another hypothesis for the prevalence of tEP in IBD patient groups could be IL-6 levels; higher circulating IL-6 levels have been associated with tEP^37^. Confounding factors were not controlled for in the study, data on smoking was unable to be obtained, and the register used did not capture any spontaneously aborted tEPs that never required medication or surgery^15^. Therefore, large-scale studies are required to estimate the true prevalence of tEP in IBD patients.

### Stratification of risk

When assessing someone in early pregnancy, there have been several attempts at scoring tools to triage patients and stratify risk. These generally combine risk factors, clinical symptoms and investigative symptoms. With regards to risk factors, a composite score can be given to known risk factors, with previous ectopic pregnancy having particular importance^38^.

## Clinical symptoms and investigative findings

### Clinical Symptoms

Tubal EP presents a wide range of symptoms, from none to profound circulatory collapse. The most common symptoms are first-trimester abdominal pain and vaginal bleeding in around two-thirds of cases. Bleeding only is more common than pain only. The bleeding is more commonly spotting or light, and the pain is more likely to be unilateral than central. However, these symptoms are common, and diagnosis cannot be made until further investigation has been performed^39^. Non-gynaecological symptoms for tEP include diarrhoea, vomiting, or dizziness and presentation of these symptoms may not initially be considered as a possible tEP and, therefore, a pregnancy test might not be carried out at first assessment^2,39^. Around 20% of women presenting with a tEP have shoulder tip pain, fainting and shock, symptoms that can be indicative of a ruptured tEP^1^. Women of reproductive age who present acutely unwell should be tested for pregnancy immediately to either confirm or refute the possibility of pregnancy underlying these symptoms. Scoring paradigms looking at adding symptoms to risk factors score significant iliac fossa pain and prolonged per vaginum (PV) spotting highest for patient triage.

### Investigation

Women often present early in the clinical course of EP with mild unilateral abdominal pain and abnormal vaginal bleeding^40^. Approximately 70% of EPs are diagnosed in the initial transvaginal ultrasound (TVS)^40^. A preliminary diagnosis of a pregnancy of unknown location (PUL) is given when TVS fails to positively identify either a normally located or EP along with a positive serum β-hCG^1^. Approximately 30% of PUL cases develop into normally located pregnancy, whereas the majority of PUL cases (50–70%) are ultimately diagnosed with a failing pregnancy, either a tEP or miscarriage^41^. An ongoing normally located pregnancy will usually be detectible in a transvaginal ultrasound when β-hCG concentration is greater than 1500 - 2000 IU/mL^42^. In PUL cases where women are most likely to have an evolving EP, a serial quantitative serum β-hCG analysis, with or without serum progesterone assessment, is adopted as a focus follow-up strategy. The expected β-hCG concentration pattern in IUP is a sharp rise in concentration over the first 4 weeks of gestation followed by a slower rise until 10 weeks. However, in PUL, the use of β-hCG discriminatory levels when an IUP should be visible by ultrasound is discouraged as it is now widely recognised that β-hCG quantification can be non-specific, and some tEPs can rupture with β-hCG concentrations below discriminatory levels^40,43^. However, the presence of an empty uterus with β-hCG concentrations above the discriminatory range without a clear history of miscarriage is concerning. In cases where a tEP is suspected but the non-invasive diagnostic tests have not been conclusive, the patient should be closely monitored with safe-guarding advice as it is possible for rupture to occur in resolving tEPs^43^.

In low-income developing countries, the early diagnosis of ectopic pregnancy remains a challenge^12,44^. Most women with tEP in developing countries present with evidence of internal bleeding or ruptured tEP, and this is associated with a lack of family planning, contraception and early pregnancy services^12^. In rural areas, ultrasound facilities may also be absent, making early diagnosis difficult^44^. Therefore, laparotomies for diagnosis and treatment are currently the commonest treatment for acutely unwell women^12,44^. Improving the availability of diagnostic tools and early, including medical, intervention in resource-poor settings may reduce deaths from ruptured tEPs.

### Predictive paradigms

Ectopic pregnancy can be difficult to diagnose, and in over half the cases, the diagnosis is not made on first consultation. Various predictive models are in use for tEP that take into consideration the risk factors, presenting symptoms and investigative findings. One model distilled 22 factors down to the 5 most significant elements of the scoring tool: a history of PID; emergency contraception; cervical tenderness; serum hCG concentration ≥1000 IU/l; and ultrasonic finding of adnexal mass^45^. However, many tEPs do not fulfil these criteria. The M6 model can be useful in women with a PUL. This is a two-step protocol where the initial step is looking at serum progesterone as a marker of previous hCG dynamics. If the level was ≤2 nmol/l, the patients were discharged with a home pregnancy test in two weeks. If serum progesterone was >2 nmol/l, an individualised plan was calculated based on serial hCG concentrations^46^.

## Clinical management of tEP

An untreated tEP has a range of outcomes, from spontaneous regression to tubal rupture^42^. There are currently three clinical management options for tEP: expectant; medical; and surgical management^47^. Expectant management is described as ‘watchful waiting’, whereas medical management is the chemotherapeutic targeting of a tEP using methotrexate, and surgical management is an invasive surgical procedure to either surgically remove the tEP or fully remove the fallopian tube containing the tEP^9,42^.

### Expectant management

In developed countries such as the UK, avoiding over-intervention of tEPs that are likely to resolve spontaneously is a significant challenge; therefore, clinicians have developed guidelines for expectant management^40,48^. Expectant management is when a patient with a confirmed tEP is at low risk of having a tubal rupture and, therefore, is closely monitored to ensure that the tEP resolves spontaneously^42^. The main criteria for expectant management are that patients are haemodynamically stable, pain-free, have a tEP measuring less than 35 mm with no visible heartbeat, have low or declining β-hCG concentrations and a low concern for tubal rupture^42,47^. Generally, the follow-up and subsequent management of patients is similar to those who have had methotrexate. There is conflicting evidence on the efficacy of expectant management in avoidance of subsequent surgery^42,49,50^. However, recent studies have suggested that expectant management is as safe and efficacious as methotrexate treatment in selected patients^49,50^.

### Medical management

Methotrexate is a chemotherapeutic agent that acts as a folate antagonist and prevents DNA replication^1,40^. It works by targeting the rapidly proliferating cells of a developing conceptus, reducing cell viability and β-hCG secretion to facilitate the resolution of a tEP^1^. Around 15% of the time, a second dose of methotrexate is required. Methotrexate is generally well tolerated; however, one in three women may experience some mild effects such as nausea, diarrhoea, and abdominal bloating^2,40^. Potential serious side effects of methotrexate treatment, such as hepatotoxicity, bone marrow toxicity and alopecia, are very rare^2^.

Serum β-hCG concentrations at first presentation of tEP have been found to be associated with treatment success^40,51^. The total success rate for methotrexate management of tEP is approximately 87%; the highest success rate has been seen in patients with β-hCG levels <1500 IU/L (90–96% success)^52^. Success rate of methotrexate significantly drops with β-hCG levels higher than 1500 IU/L and patients with a baseline β-hCG concentration of 5000 IU/L were found to be 4 times more likely to have treatment failure after a single dose of methotrexate^40,51^. Therefore, treatment centres usually offer methotrexate treatment to women who have β-hCG levels less than 3000 IU/L. Success is more likely when the hCG increment in the 48 hours before methotrexate is <12% and where hCG falls in the first four days after treatment.

Clinical trials are currently ongoing to investigate other drugs, such as combination treatment with methotrexate^53^. One such adjuvant drug is gefitinib, an epidermal growth factor (EGF) receptor-tyrosine kinase inhibitor, which works by targeting EGF, which is essential for placental development^53,54^. Phase I trials of methotrexate and gefitinib combination have aimed to show the safety, tolerability and efficacy of the treatment^53,54^. The results showed that β-hCG levels by day 7 were significantly lower than controls, and the average time for a tEP to resolve was reduced by around 34%^54^. Phase I clinical trials concluded that gefitinib in combination with methotrexate is potentially more effective than methotrexate alone in resolving tEPs. However, a recently conducted large-scale, multi-centre, nationwide, UK clinical trial of methotrexate and gefitinib in combination showed that, as well as a higher incidence of reported adverse symptoms, adjuvant gefitinib in addition to methotrexate has no clinical benefit when compared to methotrexate alone^55^. 

### Surgical management

The surgical management of tEP remains a necessary treatment for tEP, either because a woman has significant pain, live tEP, haemoperitoneum or serum hCG > 3000–5000 IU/l or when other medical management efforts have failed to resolve a tEP^40,56^. Laparoscopy has largely replaced laparotomy in the treatment of tEP; it is minimally invasive, safer, faster and cheaper^40^. In cases where a patient is presenting with a ruptured tEP and is in hypovolemic shock, a laparotomy may be necessary^9^. A tEP can be surgically removed in one of two laparoscopic techniques: conservative surgery (salpingostomy) or radical surgery (salpingectomy)^56^.

***Salpingostomy versus salpingectomy.*.** Salpingostomy is the preferred technique to conserve the fallopian tube; an incision in the Fallopian tube is made to dissect out the pregnancy tissue^40^. However, salpingostomy presents a risk of persistent trophoblast. Multiple retrospective studies have shown that persistent trophoblast rates in salpingostomy are around 3–20% compared to only 1.8% for salpingectomy^40,56^. Follow-up after salpingostomy with serial β-hCG measurements is recommended to assess the risk of ongoing trophoblast as if β-hCG concentrations fail to decline methotrexate treatment may be required^40^. Salpingectomy is the surgical removal of a fallopian tube; in cases where the contralateral tube is healthy with no tubal pathology, a salpingectomy is recommended as best practice, minimising the risk of trophoblastic persistence^40,57^.

### The management of tEP during the COVID-19 pandemic

As the COVID-19 pandemic has progressed, new data has emerged showing a detrimental impact on pregnancy outcomes during the pandemic due to lockdowns, overwhelmed healthcare services and a reluctance to seek help for symptoms^58^. Various studies from different countries have identified an increase in the presentation of ruptured tEP during the COVID-19 lockdown compared to that of pre-lockdown incidence, suggesting that this increase is lockdown specific^58–60^. One study from the US has shown that 83% of women presenting with tEP during the height of the COVID-19 pandemic were haemodynamically unstable^58^. This increase in presentation with ruptured tEP is concerning due to the increased associated morbidity and mortality. 

## Reproductive outcomes after tEP management

Clinical treatment of tEP can be fertility altering^61^ as a fallopian tube has been removed, or if not removed, potentially damaged.

### Fertility outcome after medical management

Observational studies conducted which have focussed on subsequent fertility in women who have undergone medical management for tEP have shown that the rate of new pregnancy is around 80%^62,63^. Three studies have reported remarkably similar results with regard to tubal patency rates after methotrexate treatment. There is normal ipsilateral tubal patency in 82–84% of cases. It has also been shown that methotrexate to treat ectopic pregnancy has no effect on the subsequent treatments used for infertility and that there was no significant difference in the number of oocytes collected before and after methotrexate treatment^64^. Studies have shown that only a previous history of infertility is associated with subsequent poor reproductive performance after medical management of tEPs^62,63^. The medical management of tEP with methotrexate has shown no significant differences in the rate of recurrence of tEP compared with other management methods^65^.

### Fertility outcomes after surgical management

In surgical management for the treatment of tEP, salpingectomy can reduce fertility. Although an egg released from the right side can go down the left fallopian tube, that doesn’t happen every cycle, meaning that the number of chances of pregnancy over a year would be reduced. However, most would achieve a pregnancy without fertility intervention. As guidance suggests, a salpingectomy should be performed if there is a normal contralateral fallopian tube; most cases of salpingostomy are performed whether there are known tubal problems. However, recent observational studies have shown that the subsequent rate of intrauterine pregnancy tended to be lower in patients who had undergone a salpingectomy compared to those who had undergone salpingostomy^63,65,66^. In one large study, there was a 20% conversion of salpingostomy to salpingectomy during primary surgery. In another study, the rate of conversion to salpingectomy was 15%, with a lower rate (10%) in smaller stable ectopics and a larger rate (21%) in larger symptomatic ectopics. One study looking at tubal patency rates after salpingostomy showed normal ipsilateral patency in 60% of cases, and this did not differ from those treated with methotrexate. There is also a trend for higher tEP recurrence after salpingostomy compared to salpingectomy^67^. The observational studies have focused on short-term follow-up for reproductive outcomes; further studies are required to assess long-term effects of salpingostomy and salpingectomy for tEP on reproductive health.

## Summary

Tubal ectopic pregnancy remains the leading cause of direct maternal death in the first trimester of pregnancy worldwide; therefore, early diagnosis of tEP is key to reducing maternal mortality and improving the success rates of treatments. The emergence of new risk factors for tEP is important for improving the current understanding of ectopic pregnancy as well as highlighting areas of interest in biological research. Scoring tools can help with triage of patients and highlight best management. In the UK, NICE guidelines have now been updated to include expectant management to avoid over-intervention in cases where there is a low risk of tubal rupture and the tEP is likely to resolve spontaneously. Medical management using methotrexate is safe and effective for small tEPs with no increased risk of recurrent tEP compared to surgical management. Clinical trials are currently ongoing to improve the success of the medical management of tEPs, which may reduce the risk of short- and long-term morbidity related to ectopic pregnancy. Regular assessment and research in tEP diagnosis and management is important to the continuing improvement of care, treatment and reducing mortality.
